# The Associations between Functional Fitness Test Performance and Abdominal Obesity in Healthy Elderly People: Results from the National Physical Fitness Examination Survey in Taiwan

**DOI:** 10.3390/ijerph18010264

**Published:** 2020-12-31

**Authors:** Hsin-Han Chen, Hui-Ling Chen, Yi-Tien Lin, Chaou-Wen Lin, Chien-Chang Ho, Hsueh-Yi Lin, Po-Fu Lee

**Affiliations:** 1 Graduate Institute of Technological and Vocational Education, National Taipei University of Technology, Taipei City 106, Taiwan; viva71215@gmail.com; 2 Graduate Institute of Educational Leadership and Development, Fu Jen Catholic University, New Taipei City 242, Taiwan; 024145@mail.fju.edu.tw; 3 Graduate Institute of Business Administration, Fu Jen Catholic University, New Taipei City 242, Taiwan; D00014@mail.fjuh.fju.edu.tw; 4 Department of Marine Leisure and Tourism, Taipei University of Marine Technology, Taipei City 111, Taiwan; mikelin319@mail.tumt.edu.tw; 5 Department of Physical Education, Fu Jen Catholic University, New Taipei City 242, Taiwan; 093703@mail.fju.edu.tw; 6 Research and Development Center for Physical Education, Health, and Information Technology, College of Education, Fu Jen Catholic University, New Taipei City 242, Taiwan; 7 Office of Physical Education, Fu Jen Catholic University, New Taipei City 242, Taiwan; 8 Department of Leisure Industry and Health Promotion, National Ilan University, Ilan County 260007, Taiwan; hilin@niu.edu.tw; 9 Graduate Institute of Sport Coaching Science, Chinese Culture University, Taipei City 111, Taiwan

**Keywords:** functional capability, independent ability, success aging, abdominal obesity

## Abstract

The relationships between different functional fitness performance and abdominal obesity among the elderly have not been widely discussed in the literature. The present study aimed to investigate the associations between functional fitness test performance and abdominal obesity in Taiwanese elderly people. A total of 22,399 items of data from the National Physical Fitness Survey Databases in Taiwan (NPFSD 2014-15) were reviewed and analyzed. The quartiles of functional fitness test results were identified as the dependent variable in the multiple linear regression analysis to examine the association between functional fitness and abdominal obesity status. The results showed that body balance (odds ratios (ORs) listed from 1.18 to 2.29, *p* < 0.05) and flexibility (ORs listed from 1.23 to 2.16, *p* < 0.05) were critically associated with abdominal obesity. However, measurements related to muscle strength revealed the limited effect sizes for abdominal obesity. From a disability prevention perspective, the development of muscle strength in the elderly should be emphasized and encouraged to maintain their daily capabilities instead of satisfaction with a lean body.

## 1. Introduction

Body composition modifications are crucial for successful aging. Elderly populations are usually characterized by increased body fat mass and decreased muscle mass and bone density compared to younger adults [1]. Moreover, distribution of body adiposity may change with age. A recent study indicated that body adiposity is generally increasing in the abdominal area and mainly with a reduction in subcutaneous fat in late life [2]. In fact, abdominal obesity has been reported for its significant association with metabolic syndrome and involvement with other health-related risk factors, such as blood lipid disorders, inflammation, insulin resistance, and diabetes, which result in negative impacts on society [3]. Specifically, abdominal obesity is correlated with higher cardiovascular disease rate (CVD) [4] and increased risks of all-cause mortality [5], and it is also associated with different types of cancers [6,7] among elderly people. Previous studies have proposed the impacts of abdominal obesity and suggested immediate attention being paid to this problem, especially in the context of Taiwan having recently reported that obesity prevalence has sharply increased from 11.8% to 22.0% in the past 10 years [8]. 

Furthermore, abdominal obesity has previously been reported as a risk factor for disability in elderly people [9]. Souza Barbosa and colleagues [10] based on a 4-year follow-up study pointed out that abdominal obesity significantly increased the risk of mobility disability within a population aged 64 to 74 years. Moreover, abdominal obesity accompanied with dynapenia (known as dynapenic abdominal obesity) significantly increase the risk of single fall among elderly people [11]. Nevertheless, a consistent finding from the previous review suggested that abdominal obesity in elderly people threatens their walking, stair-climbing, and chair-rise abilities, subsequently reducing their quality of life [12]. Although any type of obesity might negatively impact the aging process, abdominal obesity has been identified to increase the risk of disability in an elderly population and raise the costs of public healthcare [13,14]. Body composition and functional capability in late life are of significant importance for elderly people.

On the other hand, differences in sociodemographic characteristics and health-related habits may play important roles in abdominal obesity. Previous studies have indicated that abdominal obesity could be significantly associated with education level [15], monthly income [16], marital status [17], smoking status [18], betel nut chewing [19], and perceived health status [20]. In particular, education is crucial in overweight and obese status. For example, commonly, education level is not only a deciding factor in relation to available income for health-related services and products, but it also relates to information assessment and cognition skills that may influence health-related behaviors [21]. Perceptions of maintaining/improving body composition could differ as a result of many factors.

Furthermore, those with abdominal obesity may be caught in a vicious cycle, with insufficient participation in physical activity. Specifically, abdominal obesity could restrain muscle strength through inflammatory and endocrine mechanisms [22]. For example, sarcopenic obese and obese populations usually have higher intermuscular adipose tissue mass (IMAT), significantly correlated with monocyte chemoattractant protein-1, which is a novel biomarker of adipose tissue inflammation [23]. Researches have also pointed out that elderly people with high adiposity engaged in less physical activity may have an increased risk of mobility disability [24]. Thus, promoting physical activities among elderly people is crucial for them to maintain physical fitness and to avoid abdominal obesity, as well as functional limitations in their daily life [25,26,27]

Although physical activities develop one’s fitness level, there are variations among different populations and their F.I.T.T. (frequency, intensity, type, and time). Physical fitness in elderly people involves different aspects, such as cardiovascular fitness, muscle strength and endurance, flexibility, balance, and body composition. These health-related components of physical fitness according to the American College of Sports and Medicine (ACSM) are measurable by functional fitness tests that have previously been developed and validated [28]. Recently, Lee and colleagues’ [29] study investigated the association between physical fitness and abdominal obesity risk among Taiwanese adults aged 23 to 64 years. Their results suggested that muscular strength, cardio endurance, and flexibility may be effective predictors of abdominal obesity. However, to the best of our knowledge, there is a lack of discussion regarding the association between the performance of elderly people on functional fitness tests and abdominal obesity status. Particularly, which type of physical fitness (and at what level) is abdominal obesity most associated with? This question appears to remain unanswered in the literature. Therefore, the present study aimed to determine the associations between functional fitness test performance and abdominal obesity in Taiwanese elderly people.

## 2. Materials and Methods 

### 2.1. Study Design and Participants

A cross-sectional dataset from the National Physical Fitness Survey Databases in Taiwan (NPFSD 2014-15) was reviewed to determine the associations between various functional fitness test performances and abdominal obesity status in Taiwanese elderly people. This dataset was derived from and charged by the Taiwanese Sports Administration, Ministry of Education. The data from the survey serve as the deidentified dataset for policymaking and public researches. Convenience sampling was used for data collection within 46 test stations in 20 major cities or counties in Taiwan. The data were collected from October 2014 to March 2015. Data from 22,399 healthy elderly people aged 65 years and above were applied in this study. The participants who did not pass the preliminary health check were excluded from the survey (as described below). Collection of data in the NPFSD was conducted by a group of trained examiners. The examiners attended at least 24 h of official training courses and passed the relevant tests. These training courses provided knowledge of physical fitness theories as well as practical training sections in order to educate and produce qualified and capable examiners licensed to conduct the National Physical Fitness Survey in Taiwan. The purpose and the measurements of the survey were first explained to the participants. Informed consent was provided for data collection. The data acquisition, analysis procedure, and study design were reviewed and approved by the Ethical Committee of Chung Shan Medical University (CS2-16114).

### 2.2. Data Collection Protocol

The data collection procedure was mainly conducted in four stages. First, participants were preliminary checked for their resting heart rate and blood pressure, and they were also assessed by a structural safety questionnaire to ensure their safety. Second, participants without further safety concerns were then proceeded for demographic and life habit data collection. Third, the anthropometric assessments were conducted. Lastly, before the functional fitness tests, the participants were completed a 10 min static warm-up session as instructed by the examiners. Then, they were required to perform the measurements as mentioned below. Participants were provided 3 to 5 min of rest per interval. The participants were classified into four groups according to the quartiles for further comparison.

### 2.3. Measurement

#### 2.3.1. Demographic Data 

In the demographic data collection procedure, the participants were first required to provide their demographic characteristics, either by filling out a form or dictating their answers. The demographic items included their age (classified as 65–69, 70–74, 75–79, 80–84, and 85 and above years), gender, education status (elementary school or lower, junior or senior high school, and college or higher), marital status (never married, married, and divorced/separation/widowed), and monthly income (NTD 20,000 and below, NTD 20,001 to 40,000, and NTD 40,001 and above). The data regarding life habits included their smoking history (never, current, former) and betel nut chewing history (never, current, former). Moreover, participants were also requested to answer their perceived health status (excellent or good, fair, poor or very bad). These items were included to gain an understanding of whether the demographic differences exist in the study population. 

#### 2.3.2. Anthropometric Assessment

The anthropometric assessment was measured by body weight, height, waist circumference (WC), and hip circumference (HC) without shoes and heavy clothes. The scales used in the National Physical Fitness Survey were approved by the sports administration, ministry of education, Taiwan. Participants were required to maintain a center posture for the anthropometric assessments. Bodyweight was measured to the nearest 0.1 kg using a weighing scale. Body height was measured to the nearest 0.1 cm using a wall-glued metal measuring tape and an acute-angled headpiece while the subjects stood against a plumb-checked vertical wall. Body mass index (BMI) of the participants was calculated as their weight in kilograms divided by the square of their height in meters. The WC measurement (measured to the nearest 0.1 cm) was determined using a soft measuring tape at the level of the natural waist, which was identified as the level at the hollow molding of the trunk when the trunk was bent laterally. The HC measurement (measured to the nearest 0.1 cm) was determined at the greater trochanter level. The waist-to-hip ratio (WHR) was also calculated. The cut-off WC points for abdominal obesity were suggested by Taiwan’s Ministry of Health and Welfare (MOHW), including nonabdominal obesity (WC < 90 cm for men and WC < 80 cm for women) and abdominal obesity (WC ≥ 90 cm for men and WC ≥ 80 cm for women) categories.

#### 2.3.3. Functional Fitness Tests

In order to identify the functional capacity of Taiwanese elderly people, the NPFSD 2014-15 assessed five main aspects of functional fitness with nine measurements: (1) aerobic endurance—2 min step; (2) muscle strength and endurance—30 s arm curl and 30 s chair stand; (3) flexibility—back scratch and chair sit-and-reach test; (4) balance—one-leg stance with eyes open and 8-foot up-and-go; and (5) body composition (BMI and WHR). Excluding the one-leg stance with eyes open test, which was developed by another study [30], the other functional fitness tests were conducted according to the functional fitness test manual [31]. As mentioned earlier, the examiners who assessed the tests completed the official training programs for this survey. The procedures of the functional fitness tests are briefly descripted below, excluding the measurements of BMI and WHR, as these have previously been mentioned above:

(1) 2 min step: The participant steps and lifts their knees between their patella and iliac crest for 2 min. The total number of times the movement is completed in 2 min is recorded.
(2) 30 s arm curl: Participants sit on a chair and curl their dominant arm with a weight (5 pounds for females, 8 pounds for males). The arm must be fully bent and straightened at the elbow. The upper arm is braced and close to the body so that only the lower arm can move. The number of times the movement is completed in 30 s is recorded.
(3) 30 s chair stand: Participants sit in the middle of a chair and cross their hands on the opposite shoulder. Keeping their feet flat on the floor with a straight back, they repeat the full standing position and sit back down for 30 s. The number of times the movement is completed within the period is recorded.
(4) Back scratch: Participants, in the standing position, place one hand behind their head and another hand behind their back. Then, they reach as far as possible to touch the palm or overlap the middle fingers of both hands. Participants are allowed to practice 2 times before completing the test 2 times. The best score to the nearest centimeter is recorded.
(5) Chair sit-and-reach test: Participants sit on the edge of a chair. One foot remains flat on the floor, while the other leg is extended forward with a straight knee, the heel on the floor, and the ankle bent at 90 degrees. The participants cross both hands and even their middle fingers. Then, they reach forward as far as possible toward their toes. If the participants cannot reach their toes, the distance between the fingertips and toes is recoded as a negative score. In contrast, if the participant’s fingertips overlaps their toes, their overlapping distance is recorded positively.
(6) One-leg stance with eyes open: The duration for which the participants stand on one leg with their eyes open and without hopping or putting their raised foot down is recorded in seconds.
(7) 8-foot up-and-go: A marker (a cone is used) is placed 8 feet in front of a chair. Participants fully sit on the chair with their hands resting on their knees and their feet flat on the floor. The time from sitting, to moving around the cone, returning to the chair, and then back to the starting position is recorded.

### 2.4. Statistical Analyses

Data were analyzed with the Statistical Analysis System (SAS) software package (Version 9.4, SAS Institute Inc., Cary, NC, USA). Student’s *t*-test or chi-square test was conducted to compare the demographic and functional fitness performance between nonabdominal obesity and abdominal obesity populations. Significant differences between the two groups are considered as the confounders provided for the regression model adjustments in further analyses. The quartiles of functional fitness test results were identified as the dependent variable in the multiple linear regression analysis to examine the association between functional fitness and abdominal obesity status. Furthermore, the multiple logistic regression models were used to estimate the odds ratios (ORs) and 95% confidence interval (CI) to associate the quartiles of functional fitness performance with abdominal obesity risks after adjusting for potential confounders. All data were expressed as means ± standard deviation (SD) or frequency percentage. The significance level adopted to reject the null hypothesis was *p* < 0.05. The statistical analysis process in the present study has been described previously [29,32,33,34].

## 3. Results

In total, 22,399 items of data from 8017 male and 14,372 female participants were included in the present study. The results of demographic and life habit information are shown in Table 1. The population was aged from 65 years to 103 years. Women had a higher abdominal obesity rate (69%) than men (47%). Based on the results of demographic and life habit comparisons, in general, nonabdominal obese participants in both genders were higher educated, had higher rates of a higher income, reported a greater proportion of excellent or good health status, and had lower smoking rates. Moreover, men in the nonabdominal obese group had a significantly lower rate of chewing betel nut; women in the nonabdominal obese group had a higher proportion of never being married. Furthermore, variables with these significant results were applied for further analyses.

Table 2 compares the results of functional fitness performance between the abdominal and nonabdominal obese groups. Except for the results from the 30 s arm curl test, which did not reach the significant level, both men and women in the nonabdominal obese group had better performances in almost all of the functional fitness tests (i.e., 2 min step test, 30 s chair stand, back scratch test, chair sit-and-reach test, 8-foot up-and-go test, and one-leg stance with eyes open test).

The results of the multivariate logistic regression analyses are shown in Table 3 and Table 4. Confounder adjustments were also applied in these analyses. Further, the quartiles of each functional fitness tests results were used to determine the odds and the trend within each measurement. The fourth quartile data were appointed as the reference group to compare the likelihood of abdominal obesity. For the male population (Table 3), several significant ORs were found in the 2 min step test (Q3; OR: 1.21, *p*-value: 0.048), 30 s chair stand test (Q1; OR: 1.29, *p*-value: 0.026), back scratch test (Q3; OR: 1.23, *p*-value: 0.038), chair sit-and-reach test (Q1; OR: 1.72, *p*-value: < 0.001; Q2; OR: 1.30, *p*-value: 0.015; test for trend: *p*-value <0.001), 8-foot up-and-go test (Q1; OR: 0.80, *p*-value: 0.031), and one-leg stance with eyes open test (Q1; OR: 2.29, *p*-value: < 0.001). Our analyses did not find significant ORs in regard to the 30 s arm curl test in the male population. The significant odds exceeded for abdominal obesity were revealed to be from 20% to 129% when compared to the reference group. Some associations between specific functional fitness performance and abdominal obesity warrant further discussion.

On the other hand, Table 4 presents the analysis results from the female population and shows significant ORs for the 30 s arm curl test (Q2; OR: 0.81, *p*-value: 0.013), 30 s chair stand test (Q1; OR: 1.26, *p*-value: 0.015; Q2; OR: 1.24, *p*-value: 0.010; test for trend: *p*-value = 0.006), back scratch test (Q1; OR: 2.16, *p*-value: < 0.001; Q2; OR: 1.34, *p*-value: < 0.001; Q3; OR: 1.28, *p*-value: < 0.001; test for trend: *p*-value <0.001), chair sit-and-reach test (Q1; OR: 1.29, *p*-value: 0.001; Q2; OR: 1.27, *p*-value: 0.001; test for trend: *p*-value < 0.001), and one-leg stance with eyes open test (Q1; OR: 1.44, *p*-value: < 0.001; Q2; OR: 1.24, *p*-value: 0.004; Q3; OR: 1.18, *p*-value: 0.019; test for trend: *p*-value < 0.001). There were no significant ORs found for the 2 min step test or 8-foot up-and-go test in the female population. The significant odds exceeded for abdominal obesity ranged from 18% to 116% when compared to the reference group.

## 4. Discussion

The present study aimed to investigate the associations between functional fitness test performance and abdominal obesity risk among healthy Taiwanese elderly people. Our results contained the data from 22,399 Taiwanese elderly people and indicated that the intracomparison within each functional fitness measurement provided some information to be further considered. First, the lower scores on the one-leg stand with eyes open test suggested critical associations with abdominal obesity for both genders. Specifically, the ORs of the lowest quartiles were 2.29 for men and 1.44 for women. It is understandable that physically untrained populations commonly perform lower-limb and core muscle strength exercises, which are fundamental for body balancing. Previous studies have implied that functional fitness exercise training is positively associated with body balance abilities among elderly people [35]. Moreover, studies have indicated that body balance ability is negatively associated with abdominal obesity [36,37]. The present study has similar results; notably, the male population appeared to have a considerable effect size in the relationship. In other words, when compared with the reference group (one-leg stance score > 30 s), participants in the lowest scores (one-leg stance score < 5 s) had an exceeded likelihood of 129% to have abdominal obesity.

On the other hand, flexibility might also be considerably associated with abdominal obesity. For example, the chair sit-and-reach tests in men and the back scratch tests in women provided more significant results regarding abdominal obesity. The highest OR for the chair sit-and-reach test in men was 1.72, and the highest OR for the back scratch test was 2.16 in women. Although one of the quartiles did not reach significance, the trends were critically performed to identify trend associations. Specifically, both male and female results showed that body flexibility could be associated with higher abdominal obesity risks, and the highest odds were 2.16 in females (back scratch). Accordingly, the correlation between abdominal obesity and metabolic syndrome has been reported [38], and Vieira and colleagues [39] also suggested that elderly people with metabolic syndrome have significantly lower scores on body flexibility tests than those without it. Thus, body flexibility performance might be physically obstructed by adipose tissue mass. The tests, such as the sit-and-reach test, may be unable to reflect the lower-limb muscle flexibility among abdominal obese elderly people. Alternative approaches should be developed for such a population.

Interestingly, the associations between abdominal obesity and other functional fitness measurements seem to present minor or limited effects. Specifically, the exceeded likelihoods for abdominal obesity were listed from 19% to 26%. Measurements of upper-limb muscle strength in males and mobility and cardiovascular fitness in females even appeared to have no significant effects on abdominal obesity. However, some of our results are supported by previous studies. For example, Dulac and colleagues [40] recently observed a significant correlation between waist circumference and chair stand test performance among elderly females.

Nevertheless, their study pointed out that functional fitness may not be associated with abdominal obesity but rather lower-limb muscle strength. Although it is known that decreasing physical fitness inevitably accompanies the aging process [41,42], muscle strength can postpone the speed of this process of aging and maintain functional abilities among elderly people. The functional fitness measurements are more likely to be associated with muscle strength performance [43]. In order to maintain the ability to live independently, elderly people should mainly focus on their muscle strength level to avoid sarcopenia rather than seeking a lean body [44]. 

The strength of this study was the use of a representative dataset which provided satisfactory results. However, there are some limitations and future suggestions that should be addressed. First, the present study mainly focused on a Chinese–Taiwanese elderly population. However, a previous study has pointed out that the prevalence of abdominal obesity could be variable among different ethnicities [45]. A race/ethnicity perspective on the relationship between functional fitness and abdominal obesity has yet to be discussed. Future studies should investigate populations from different races/ethnicity for a comprehensive understanding. Second, using WC to indicate abdominal obesity status may reveal some measurement errors [46]. For example, the variations among different examiners are difficult to assess. Future studies may use other approaches such as dual-energy X-ray absorptiometry (DXA) to measure the exact body composition in abdominal adiposity mass, or choose other anthropometric measurements for abdominal obesity (e.g., waist-to-hip ratio, WHR). Third, due to the fact that a secondary database was applied, it was not possible to analyze other confounders such as nutrition intake or daily energy expenditure [47]. Moreover, the quality of data collection could not be guaranteed, despite the fact that this data collection process was supervised by a group of experts and government authorities. Furthermore, using *p*-values to identify the confounders could generate statistical bias in a large sample size [48]. Finally, due to the cross-sectional nature of this study, no cause-and-effect relationship can be guaranteed. Future studies should be conducted with a longitudinal study design in order to provide clinical importance of functional fitness for abdominal obesity risk.

## 5. Conclusions

In conclusion, according to the results of this study, body balance and flexibility are associated with abdominal obesity the most among the Taiwanese elderly population. Other muscular strength performances had limited effects on abdominal obesity. Development of muscle strength in the elderly people should be emphasized and encouraged to maintain their functional abilities.

## Figures and Tables

**Table 1:** Demographic characteristics and life habits of the study population.

Variables	Men (*N* = 8017)			Women (*N* = 14,372)		
	Abdominal Obesity (*n* = 3780)	Nonabdominal Obesity (*n* = 4237)	*p*-Value	Abdominal Obesity (*n* = 9880)	Nonabdominal Obesity (*n* = 4492)	*p*-Value
Age (years)			0.001 *			<0.001 *
65–69	28.12	31.25		34.72	41.90	
70–74	24.63	25.58		27.88	27.96	
75–79	23.39	20.04		21.26	17.76	
80–84	14.21	13.55		11.16	8.82	
≥85	9.6	9.58		4.98	3.56	
Height (cm)	164.06 ± 5.86	162.77 ± 6.07	<0.001 *	152.77 ± 5.82	152.50 ± 5.60	0.009 *
Body weight (kg)	71.39 ± 6.93	60.66 ± 7.33	<0.001 *	60.70 ± 7.96	51.28 ± 6.40	<0.001 *
BMI (kg/m^2^)	26.56 ± 2.38	22.88 ± 2.43	<0.001 *	26.02 ± 3.14	22.06 ± 2.53	<0.001 *
WC (cm)	96.33 ± 5.08	82.27 ± 5.40	<0.001 *	89.34 ± 6.96	74.08 ± 4.09	<0.001 *
HC (cm)	100.06 ± 4.79	92.84 ± 4.65	<0.001 *	98.37 ± 5.91	90.95 ± 4.68	<0.001 *
WHR	0.96 ± 0.05	0.89 ± 0.05	<0.001 *	0.91 ± 0.06	0.82 ± 0.05	<0.001 *
Education level (%)			<0.001 *			<0.001 *
Elementary school or lower	48.62	39.29		68.94	54.11	
Junior or senior school	30.71	34.78		23.99	32.59	
College or higher	20.67	25.93		7.07	13.30	
Income level (%)			0.004 *			<0.001 *
≤NTD 20,000	80.57	77.52		91.79	87.25	
NTD 20,001–40,000	10.83	11.76		5.19	8.17	
≥NTD 40,001	8.60	10.72		3.02	4.58	
Marital status (%)			0.612			<0.001 *
Never married	58.60	58.94		47.64	52.06	
Married	30.50	29.63		25.24	25.62	
Divorced/separation/widowed	10.90	11.42		27.12	22.33	
Self-reported health status (%)			0.003 *			<0.001 *
Excellent or good	66.61	69.76		62.63	65.89	
Fair	25.69	24.14		28.01	27.34	
Poor or very bad	7.69	6.10		9.36	6.77	
Smoking status (%)			<0.001 *			0.011 *
Never	78.60	82.11		97.19	97.97	
Current	11.62	10.70		1.98	1.26	
Former	9.78	7.19		0.83	0.77	
Chewing betel nut			<0.001 *			0.090
Never	93.37	96.26		98.79	99.21	
Current	2.61	1.12		0.81	0.51	
Former	4.02	2.63		0.40	0.28	

Abbreviations: BMI, body mass index; HC, hip circumference; NTD, new Taiwan dollar; SD, standard deviation; WC, waist circumference; WHR, waist-to-hip ratio. Values are expressed as means ± SD. Abdominal obesity, WC ≥ 90/80 cm; nonabdominal obesity, WC < 90/80 cm. * *p* < 0.05.

**Table 2:** Comparisons between abdominal obesity status and functional fitness performance.

Variables	Men (*N* = 8017)			Women (*N* = 14,372)		
	Abdominal Obesity (*n* = 3780)	Nonabdominal Obesity (*n* = 4237)	*p*-Value	Abdominal Obesity (*n* = 9880)	Nonabdominal Obesity (*n* = 4492)	*p*-Value
2 min step test (step; cardiovascular fitness)	85.17 ± 23.84	89.19 ± 23.14	<0.001 *	83.16 ± 24.19	88.29 ± 23.22	<0.001 *
30 s arm curl test (rep; muscle strength and endurance)	17.56 ± 5.48	17.78 ± 5.77	0.077	17.24 ± 5.45	17.23 ± 5.58	0.873
30 s chair stand test (rep; muscle strength and endurance)	14.52 ± 4.70	15.72 ± 4.93	<0.001 *	14.17 ± 4.53	15.58 ± 4.71	<0.001 *
Back scratch test (cm; flexibility)	−13.94 ± 12.82	−8.71 ± 12.35	<0.001 *	−6.57 ± 11.09	−1.28 ± 9.34	<0.001 *
Chair sit-and-reach test (cm; flexibility)	1.74 ± 8.25	3.37 ± 8.35	<0.001 *	4.90 ± 7.84	6.55 ± 8.11	<0.001 *
8-foot up-and-go test (s) (s; balance)	7.47 ± 1.88	6.90 ± 1.84	<0.001 *	7.64 ± 1.84	6.96 ± 1.72	<0.001 *
One-leg stance with eyes open test (s; balance)	13.82 ± 11.11	17.50 ± 11.66	<0.001 *	13.05 ± 10.77	17.28 ± 11.39	<0.001 *

Abbreviations: SD, standard deviation. Values are expressed as means ± SD. Abdominal obesity, WC ≥ 90/80 cm; nonabdominal obesity, WC < 90/80 cm. * *p* < 0.05.

**Table 3:** Multivariate-adjusted odds ratios (ORs) for abdominal obesity in relation to quartiles of health-related physical fitness measurements after adjustment for potential confounders.

Variables	Model 1 (Unadjusted)			Model 2 (Adjusted ^a^)		
	OR	95% CI	*p*-Value	OR	95% CI	*p*-Value
Men						
2 min step test (step; cardiovascular fitness)						
<73 (*n* = 2221)	1.10	0.95–1.28	0.185	1.22	0.99–1.49	0.064
73–89 (*n* = 1929)	1.10	0.96–1.27	0.183	1.13	0.93–1.39	0.219
90–103 (*n* = 1980)	1.13	0.98–1.29	0.086	1.21	1.00–1.46	0.048 *
>103 (*n* = 1887)	1.00	—	—	1.00	—	—
Test for trend	0.343			0.154		
30 s arm curl test (rep; muscle strength and endurance)						
<14 (*n* = 2071)	0.76	0.65–0.88	<0.001 *	1.10	0.89–1.35	0.393
14–17 (*n* = 1969)	0.92	0.79–1.06	0.255	1.23	0.99–1.51	0.058
18–22 (*n* = 2207)	0.96	0.83–1.10	0.538	1.14	0.94–1.39	0.188
>22 (*n* = 1597)	1.00	—	—	1.00	—	—
Test for trend	<0.001 *			0.394		
30 s chair stand test (rep; muscle strength and endurance)						
<12 (*n* = 2002)	1.22	1.04–1.43	0.013 *	1.29	1.03–1.61	0.026 *
12–14 (*n* = 1961)	1.22	1.05–1.42	0.009 *	1.12	0.91–1.39	0.288
15–18 (*n* = 2197)	1.14	0.99–1.31	0.054	1.06	0.88–1.29	0.542
>18 (*n* = 1857)	1.00	—	—	1.00	—	—
Test for trend	0.020 *			0.027 *		
Back scratch test (cm; flexibility)						
<−21 (*n* = 1991)	2.96	2.55–3.43	<0.001 *	1.21	0.98–1.50	0.080
−21–−11 (*n* = 2095)	2.19	1.90–2.53	<0.001 *	1.19	0.97–1.46	0.087
−10–0 (*n* = 2301)	1.80	1.56–2.06	<0.001 *	1.23	1.01–1.49	0.038 *
>0 (*n* = 1625)	1.00	—	—	1.00	—	—
Test for trend	<0.001 *			0.168		
Chair sit-and-reach test (cm; flexibility)						
<−2 (*n* = 2595)	1.23	1.08–1.41	0.003 *	1.72	1.42–2.08	<0.001 *
−2–0 (*n* = 1525)	1.06	0.91–1.23	0.437	1.30	1.05–1.61	0.015 *
1–8 (*n* = 2237)	1.02	0.89–1.17	0.749	1.14	0.94–1.38	0.181
>8 (*n* = 1643)	1.00	—	—	1.00	—	—
Test for trend	<0.001 *			<0.001 *		
8-foot up-and-go test (s; balance)						
>8.3 (*n* = 1845)	1.33	1.16–1.54	<0.001 *	1.25	1.02–1.54	0.031 *
7.0–8.3 (*n* = 1910)	1.36	1.19–1.55	<0.001 *	1.28	1.06–1.55	0.010 *
5.8–6.9 (*n* = 1960)	1.24	1.09–1.41	0.001 *	1.13	0.94–1.36	0.197
<5.8 (*n* = 2289)	1.00	—	—	1.00	—	—
Test for trend	<0.001 *			0.009 *		
One-leg stance with eyes open test (s; balance)						
<5.0 (*n* = 1973)	1.78	1.29–2.46	<0.001 *	2.29	1.44–3.65	<0.001 *
5.0–12.6 (*n* = 2034)	1.39	1.01–1.91	0.043 *	1.52	0.96–2.40	0.075
12.7–30.0 (*n* = 3813)	1.13	0.83–1.54	0.449	1.44	0.92–2.26	0.109
>30.0 (*n* = 193)	1.00	—	—	1.00	—	—
Test for trend	<0.001 *			<0.001 *		

Abbreviations: BMI, body mass index; CI, confidence interval; OR, odds ratio. * *p* < 0.05. ^a^ Adjusted for age, BMI, education, monthly income, self-reported health status, smoking status, and chewing betel nuts.

**Table 4:** Multivariate-adjusted ORs for abdominal obesity in relation to quartiles of health-related physical fitness measurements after adjustment for potential confounders.

Variables	Model 1 (Unadjusted)			Model 2 (Adjusted ^a^)		
	OR	95% CI	*p*-Value	OR	95% CI	*p*-Value
Women						
2 min step test (step; cardiovascular fitness)						
<70 (*n* = 4059)	1.08	0.96–1.21	0.224	1.04	0.89–1.22	0.603
70–87 (*n* = 3418)	1.02	0.91–1.14	0.772	1.12	0.97–1.30	0.129
88–101 (*n* = 3481)	0.99	0.89–1.10	0.886	1.12	0.98–1.29	0.103
>101 (*n* = 3414)	1.00	—	—	1.00	—	—
Test for trend	0.286			0.535		
30 s arm curl test (rep; muscle strength and endurance)						
<14 (*n* = 3901)	0.72	0.64–0.80	<0.001 *	0.88	0.75–1.02	0.089
14–16 (*n* = 2539)	0.72	0.63–0.81	<0.001 *	0.81	0.69–0.96	0.013 *
17–21 (*n* = 4493)	0.86	0.77–0.96	0.005 *	0.97	0.84–1.11	0.618
>21 (*n* = 3072)	1.00	—	—	1.00	—	—
Test for trend	<0.001 *			0.040 *		
30 s chair stand test (rep; muscle strength and endurance)						
<11 (*n* = 2863)	1.44	1.26–1.66	<0.001 *	1.26	1.05–1.51	0.015 *
11–13 (*n* = 3457)	1.39	1.23–1.58	<0.001 *	1.24	1.05–1.47	0.010 *
14–18 (*n* = 5312)	1.25	1.13–1.39	<0.001 *	1.11	0.97–1.27	0.144
>18 (*n* = 2740)	1.00	—	—	1.00	—	—
Test for trend	<0.001 *			0.006 *		
Back scratch test (cm; flexibility)						
<−12 (*n* = 3502)	3.82	3.38–4.33	<0.001 *	2.16	1.83–2.56	<0.001 *
−12–−2 (*n* = 3650)	2.56	2.29–2.86	<0.001 *	1.34	1.16–1.55	<0.001 *
−1–3 (*n* = 4404)	1.62	1.47–1.79	<0.001 *	1.28	1.12–1.46	<0.001 *
>3 (*n* = 2811)	1.00	—	—	1.00	—	—
Test for trend	<0.001 *			<0.001 *		
Chair sit-and-reach test (cm; flexibility)						
<0 (*n* = 2977)	0.98	0.87–1.10	0.742	1.29	1.10–1.51	0.001 *
0–3 (*n* = 4379)	1.15	1.03–1.28	0.010 *	1.27	1.10–1.46	0.001 *
4–11 (*n* = 3888)	1.06	0.95–1.18	0.288	1.13	0.99–1.30	0.079
>11 (*n* = 3104)	1.00	—	—	1.00	—	—
Test for trend	0.654			<0.001 *		
8-foot up-and-go test (s; balance)						
>8.6 (*n* = 3270)	1.42	1.26–1.61	<0.001 *	1.16	0.99–1.37	0.072
7.1–8.6 (*n* = 3491)	1.37	1.23–1.53	<0.001 *	1.12	0.97–1.29	0.128
6.0 –7.0 (*n* = 3806)	1.16	1.05–1.28	0.004 *	1.00	0.87–1.14	0.938
<6.0 (*n* = 3772)	1.00	—	—	1.00	—	—
Test for trend	<0.001 *			0.007 *		
One-leg stance with eyes open test (s; balance)						
<4.4 (*n* = 3590)	1.75	1.56–1.97	<0.001 *	1.44	1.22–1.69	<0.001 *
4.4–10.7 (*n* = 3589)	1.50	1.35–1.67	<0.001 *	1.24	1.07–1.43	0.004 *
10.8–27.0 (*n* = 3610)	1.33	1.20–1.48	<0.001 *	1.18	1.03–1.35	0.019 *
>27.0 (*n* = 3577)	1.00	—	—	1.00	—	—
Test for trend	<0.001 *			<0.001 *		

Abbreviations: BMI, body mass index; CI, confidence interval; OR, odds ratio. * *p* < 0.05. ^a^ Adjusted for age, BMI, education, monthly income, marital status, self-reported health status, and smoking status.

## Data Availability

All relevant materials are provided in the present manuscript. The raw data is allowing to be applied and is supervised by the Sports Cloud: Information and Application Research Center of Sports for All, Sport Administration, Ministry of Education, Taiwan.

## References

[1] Baumgartner R.N., Stauber P.M., McHugh D., Garry P.J. (1995). Cross-sectional age-differences in body composition in persons 60+ years of age. J. Gerontol. A Biol. Sci. Med Sci..

[2] Ponti F., Santoro A., Mercatelli D., Gasperini C., Conte M., Martucci M., Sangiorgi L., Franceschi C., Bazzocchi A. (2020). Aging and imaging assessment of body composition: From fat to facts. Front. Endocrinol..

[3] Després J.P., Lemieux I. (2006). Abdominal obesity and metabolic syndrome. Nature.

[4] Fan H., Li X., Zheng L., Chen X., Ian Q., Wu H., Ding X., Qian D., Shen Y., Yu Z. (2016). Abdominal obesity is strongly associated with cardiovascular disease and its risk factors in elderly and very elderly community-dwelling Chinese. Sci. Rep..

[5] David C.N., Mello R.B., Bruscato N.M., Moriguchi E.H. (2017). Overweight and abdominal obesity association with all-cause and cardiovascular mortality in the elderly aged 80 and over: A cohort study. J. Nutr. Health Aging.

[6] Dong Y., Zhou J., Zhu Y., Luo L., He T., Hu H., Liu H., Zhang Y., Luo D., Xu S. (2017). Abdominal obesity and colorectal cancer risk: Systematic review and meta-analysis of prospective studies. Biosci. Rep..

[7] Hidayat K., Du X., Chen G., Shi M., Shi B. (2016). Abdominal obesity and lung cancer risk: Systematic review and meta-analysis of prospective studies. Nutrients.

[8] Chang H.C., Yang H.C., Chang H.Y., Yeh C.J., Chen H.H., Huang K.C., Pan W.H. (2017). Morbid obesity in Taiwan: Prevalence, trends, associated social demographics, and lifestyle factors. PLoS ONE.

[9] Corona L.P., Silva Alexandre T., Oliveira Duarte Y.A., Lebrao M.L. (2017). Abdominal obesity as a risk factor for disability in Brazilian older adults. Public Health Nutr..

[10] Souza Barbosa F.J., Gomes C.S., Costa V.J., Ahmed T., Zunzunegui M.V., Curcio C.L., Gomez F., Guerra R.O. (2018). Abdominal obesity and mobility disability in older adults: A 4-year follow-up of the international mobility in aging study. J. Nutr. Health Aging.

[11] Oliverira Maximo R., Santos J.L.F., Perracini M.R., Oliveira C., Oliverira Duarte Y.A., Silva Alexandre T. (2019). Abdominal obesity, dynapenia and dynapenic-abdominal obesity as factors associated with falls. Braz. J. Phys. Ther..

[12] Vincent H.K., Vincent K.R., Lamb K.M. (2010). Obesity and mobility disability in the older adult. Obes. Rev..

[13] World Health Organization (2016). Global Report on Diabetes.

[14] Delmonico M.J., Harris T.B., Visser M., Park S.W., Conroy M.B., Velasquex-Mieyer P., Boudreau R., Manini T.M., Nevitt M. (2009). Longitudinal study of muscle strength, quality, and adipose tissue infiltration. Am. J. Clin. Nutr..

[15] Sardinha L.B., Santos D.A., Silva A.M., Coelho-e-Silva M.J., Raimundo A.M., Moreira H., Santos R., Vale S., Baptista F., Mota J. (2012). Prevalence of overweight, obesity, and abdominal obesity in a representative sample of Portuguese adults. PLoS ONE.

[16] Zhang H., Xu H., Song F., Xu W., Pallard-Borg S., Qi X. (2017). Relation of socioeconomic status to overweight and obesity: A large population-based study of Chinese adults. Ann. Hum. Biol..

[17] Tzotzas T., Vlahavas G., Papadopoulou S.K., Kapantais E., Kaklamanou D., Hassapidou M. (2010). Marital status and educational level associated to obesity in Greek adults: Data from the National Epidemiological Survey. BMC Public Health.

[18] Hansson P.O., Eriksson H., Welin L., Svardsudd K., Vilhelmsen L. (1999). Smoking and abdominal obesity. Arch. Intern. Med..

[19] Lin W.Y., Pi-Sunyer F.X., Liu C.S., Li T.C., Li C.L., Huang C.Y., Lin C.C. (2009). Betel nut chewing is strongly associated with general and central obesity in Chinese male middle-aged adults. Obesity.

[20] Wu S., Wang R., Jiang A., Ding Y., Wu M., Ma X., Zhao Y., He J. (2014). Abdominal obesity and its association with health-related quality of life in adults: A population-based study in five Chinese cities. Health Qual. Life Outcomes.

[21] Yu Y. (2011). Educational differences in obesity in the United States: A closer look at the trends. Obesity.

[22] Schaap L.A., Koster A., Visser M. (2013). Adiposity, muscle mass, and muscle strength in relation to functional decline in older persons. Epidemiol. Rev..

[23] Lim J.P., Chong M.S., Tay L., Yang Y.X., Leung B.P., Yeo A., Yew S., Tan C.H., Lim W.S. (2019). Inter-muscular adipose tissue is associated with adipose tissue inflammation and poorer functional performance in central adiposity. Arch. Gerontol. Geriatr..

[24] Koster A., Patel K.V., Visser M., van Eijk J.T.M., Kanaya A.M., Rekeneire N.D., Newman A.B., Tyla sky F.A., Kritchevsky S.B., Harris T.B. (2008). Joint effects of adiposity and physical activity on incident mobility limitation in older adults. J. Am. Geriatr. Soc..

[25] Henwood T.R., Taaffe D.R. (2006). Short-term resistance training and the older adult: The effect of varied programmes for the enhancement of muscle strength and functional performance. Clin. Physiol. Funct. Imaging.

[26] Paterson D.H., Warburton D.E. (2010). Physical activity and functional limitations in older adults: A systematic review related to Canada’s Physical Activity Guidelines. Int. J. Behav. Nutr. Phys. Act..

[27] McAuley E., Konopack J.F., Morris K.S., Motl R.W., Hu L., Doerksen S.E., Rosengren K. (2006). Physical activity and functional limitations in older women: Influence of self-efficacy. J. Gerontol. Ser. B Psychol. Sci. Soc. Sci..

[28] Rikli R., Jones C.J. (2013). Development and validation of criterion-referenced clinically relevant fitness standards for maintaining physical independence in later years. Gerontologist.

[29] Lee P.F., Ho C.C., Kan N.W., Yeh D.P., Chang Y.C., Li Y.J., Tseng C.Y., Hsieh X.Y., Chiu C.H. (2020). The association between physical fitness performance and abdominal obesity risk among Taiwanese adults: A cross-sectional study. Int. J. Environ. Res. Public Health.

[30] Jonsson E., Seiger A., Hirschfeld H. (2004). One-leg stance in healthy young and elderly adults: A measure of postural steadiness?. Clin. Biomech..

[31] Rikil R., Jones C.J. (2012). Senior Fitness Test Manual.

[32] Lee P.F., Ho C.C., Yeh D.P., Hung C.T., Chang Y.C., Liu C.C., Tseng C.Y., Hsieh X.Y. (2020). Cross-sectional associations of physical fitness performance level and sleep duration among older adults: Results from the national physical fitness survey in Taiwan. Int. J. Environ. Res. Public Health.

[33] Lin Y.T., Lee P.F., Lee T.S., Ho C.C. (2020). Poor physical fitness performance as a predictor of general adiposity in Taiwanese adults. Int. J. Environ. Res. Public Health.

[34] Chen H.L., Lee P.F., Chang Y.C., Hsu F.S., Tseng C.Y., Hsieh X.Y., Ho C.C. (2020). The association between physical fitness performance and subjective happiness among Taiwanese adults. Int. J. Environ. Res. Public Health.

[35] Lee H.C., Lee M.L., Kim S.R. (2015). Effect of exercise performance by elderly women on balance ability and muscle function. J. Phys. Ther. Sci..

[36] Jeon B.J. (2013). The effects of obesity on fall efficacy in elderly people. J. Phys. Ther. Sci..

[37] Hassinen M., Komulainen P., Lakka T.A., Vaisanen S.B., Rauramaa R. (2005). Associations of body composition and physical activity with balance and walking ability in the elderly. J. Phys. Act. Health.

[38] Cabrera M.A., Gebara O.C., Diament J., Nussbacher A., Rosano G., Wajngarten M. (2007). Metabolic syndrome, abdominal obesity, and cardiovascular risk in elderly women. Int. J. Cardiol..

[39] Vieira D.C.L., Tibana R.A., Tajra V., Nascimento D.C., Farias D.L., Oliveira Silva A., Teixeira T.G., Fonseca R.M.C., Oliveira R.J., dos Santos Mendes F.A. (2013). Decreased functional capacity and muscle strength in elderly women with metabolic syndrome. Clin. Interv. Aging.

[40] Dulac M.C., Carvalho L.P., Aubertin-Leheudre M. (2018). Functional capacity depends on lower limb muscle strength rather than on abdominal obesity in active postmenopausal women. Menopause.

[41] Riebe D., Blissmer B.J., Greaney M.L., Garber C.E., Lees F.D., Clark P.G. (2009). The relationship between obesity, physical activity, and physical function in older adults. J. Aging Health.

[42] Tuna H.D., Edeer A.O., Malkoc M., Aksakoglu G. (2009). Effect of age and physical activity level on functional fitness in older adults. Eur. Rev. Aging Phys. Act..

[43] Kostic R., Pantelic S., Uzunovic S., Djuraskovic R. (2011). A comparative analysis of the indicators of the functional fitness of the elderly. Facta Univ. Ser. Phys. Educ. Sport.

[44] Lee D.C., Shook R.P., Drenowatz C., Blair S.N. (2016). Physical activity and sarcopenic obesity: Definition, assessment, prevalence and mechanism. Future Sci. OA.

[45] He J., Guo S., Liu J., Zhang M., Ding Y., Zhang J., Li S., Xu S., Niu Q., Guo H. (2014). Ethnic differences in prevalence of general obesity and abdominal obesity among low-income rural Kazakh and Uyghur adults in far western China and implications in preventive public health. PLoS ONE.

[46] Dhaliwal S.S., Welborn T.A. (2009). Measurement error and ethnic comparisons of measures of abdominal obesity. Prev. Med..

[47] Ito T., Kawakami R., Tanisawa K., Miyawaki R., Ishii K., Torii S., Suzuki K., Sakamoto S., Muraoka I., Oka K. (2019). Dietary patterns and abdominal obesity in middle-aged and elderly Japanese adults: Waseda alumin’s sports, exercise, daily activity, sedentariness and health sudy (WASEDA’s Health Study). Nutrition.

[48] Lin M., Lucas H.C., Shmueli G. (2013). Research commentary-too big to fail: Large samples and the p-value problem. Inf. Syst. Res..

